# Socio-economic inequalities in access to COVID-19 tests in France in 2020: evidence from the EPICOV socio-epidemiological cohort

**DOI:** 10.3389/fpubh.2024.1434370

**Published:** 2025-01-23

**Authors:** Pierre-Yves Geoffard, Florence Jusot, Antoine Sireyjol, Josiane Warszawski, Nathalie Bajos

**Affiliations:** ^1^Paris School of Economics, Paris, France; ^2^CNRS, Paris, France; ^3^PSL Research University, Université Paris Dauphine, LEDA, UMR CNRS, Paris, France; ^4^IRIS, Inserm/EHESS/CNRS, Aubervilliers, France; ^5^CESP UMR, Université Paris-Saclay, APHP, le Kremlin-Bicêtre, France

**Keywords:** COVID-19, socio-economic inequalities, testing, random survey, France

## Abstract

**Background:**

Testing for COVID-19 has been strongly recommended for individuals experiencing COVID-19-like symptoms or those with a close relative who tested positive. In France, tests were free of charge until mid-October 2021 and became widely available after June 2020. Our main objective was to investigate whether access to COVID-19 testing in France was associated with socio-economic conditions, considering gender and ethno-racial status.

**Methods:**

A random population-based cohort survey was conducted in France in May 2020 and November 2020, including 95,388 participants aged 18 and over. We used logistic regressions to identify how having been tested in 2020 was associated with socio-economic status and exposure factors among two groups of individuals. The first group consisted of individuals who had no close relative test positive but reported experiencing COVID-19-like symptoms, such as cough, fever, dyspnea, or sudden onset of ageusia, dysgeusia, or anosmia (*N* = 12,729). The second group included individuals, with or without symptoms, who reported that a close relative had tested positive for COVID-19 (*N* = 5,360).

**Findings:**

In both groups, testing was more frequent among individuals living in urban areas. For individuals who had no close relative test positive but reported COVID-19-like symptoms, women were 1.04 (95%CI [1.01–1.06]) more likely than men to be tested, and testing decreased with age up to 35. Individuals holding a university degree were (1.08 [1.04–1.12]) more likely than those who only completed high school to be tested, as well as those in one of the three top income deciles with reference to the bottom decile (OR 1.07, [1.02–1.13] for the top decile). Ethno-racial status was not significantly associated with testing. For respondents who had a close relative test positive, testing was strongly associated with having experienced some symptoms, especially after September 1 (OR 1.34, 95%CI [1.30–1.39]). However, there was no link between testing and income, education, or ethno-racial status.

**Interpretation:**

When an individual experiences symptoms but has no close relative who is positive, the motivation to test to avoid transmitting the virus to relatives by isolating oneself is particularly strong. This strategy makes sense when there are actual possibilities to isolate, which may explain why higher income and education positively impact the propensity to test. For individuals who had a close relative test positive, most socio-economic variables were no longer related to testing once infection risk was controlled for. The availability of tests at no cost is not sufficient in itself to eliminate socio-economic inequalities in testing.

## Introduction

In an epidemic context, effective clinical management and outbreak control hinge on the detection of as many cases as possible. As early as March 2, 2020, the WHO emphasized that “Rapid collection and testing of appropriate specimens from patients meeting the suspect case definition for COVID-19 is a priority for clinical management and outbreak control” (1). Indeed, testing is the cornerstone of the test-treat-track strategy to help contain the COVID-19 pandemic. The decision to test an individual should be based solely on an assessment of the likelihood of infection, independent of sex, income, education, or other socio-economic characteristics. However, if certain social groups do not have equal access to tests, this raises significant concerns regarding both equity and outbreak control. Investigating this issue empirically is crucial, particularly as socio-economic inequalities have played a secondary role in the design of policy interventions against COVID-19 (2).

Few studies have examined socio-economic inequalities in SARS-CoV-2 testing. Although resource shortages, documented in various contexts, likely affected different socio-economic groups differently (3), assessing inequalities in access to testing has been challenging due to insufficient data (4). Some studies, using surveillance data matched with local socio-economic information, revealed lower testing uptake among minority ethno-racial groups and residents of deprived areas in Liverpool (5), undocumented migrants and homeless individuals in Geneva (6), less affluent communities in Massachusetts (7), and in Switzerland (8) and France (9). Interestingly, both Riou et al. and Vandentorren et al., though in different contexts, found that raw testing rates were actually higher among the most deprived groups; only after adjustments (for age, sex, state, and wave for Riou et al.; for population density for Vandentorren et al.) did it become apparent that access to testing was lower among those more exposed to infection risk. To our knowledge, Trevisan et al. (10) is the only study using survey data, conducted in Italy, and it found that older age was associated with lower access to testing but did not document other inequalities.

Surveillance systems do not collect data on socio-economic variables at the individual level and instead use local information as proxies, failing to provide direct insights into individual behavior. Additionally, since only individuals who got tested are registered, it is impossible to observe those who were exposed to risk but did not get tested, even though health authorities recommended it. Using survey data collected in France, our study aims to fill this gap.

France presents an interesting case for the study of testing policies and behaviors. To eliminate financial barriers, tests were free of charge and fully covered by social security for all residents. However, at the onset of the pandemic in March 2020, test supplies were very limited, and during the first half of 2020, testing was restricted to health professionals and symptomatic patients with a medical prescription. The total number of daily tests remained low, below one per thousand people.^1^ To increase supply, biological labs were offered a generous fee of 73.59€ per test. After July 24, the priority remained for symptomatic patients, but a medical prescription was no longer required. The number of tests increased steadily during summer 2020, fluctuating between 2.5 and 5.5 daily tests per thousand individuals in the fall. Testing was never mandatory but was recommended by health authorities in two situations: when a person learned that a close relative tested positive, and when a person experienced symptoms suggestive of SARS-CoV-2 infection. Disparities in testing access among these “higher risk” situations across socio-economic groups raise several issues: they contradict the ethical principle of equitable access to healthcare, hinder disease control, and may exacerbate health inequalities.

Our survey data allows us to identify individuals who have encountered one of these situations and, among them, those who have been tested or not. When a person experiences symptoms but has no known positive relative, they are at risk of both being infected and transmitting the virus to household members. A positive relative increases the salience of the virus and awareness of infection risk, but motivations for testing may vary. Testing behavior may follow different social determinants in these two scenarios.

In this paper, we analyze whether, despite the absence of out-of-pocket costs, socio-economic conditions still influence testing behaviors in France. We contrast the two situations of interest: having a close relative who is positive and experiencing symptoms without a positive relative, to detect different mechanisms at play.

## Materials and methods

### Study design and participants

The EpiCoV (Epidémiologie et Conditions de Vie) cohort was established in April 2020 with the primary aim of understanding the key epidemiological, socio-economic, and behavioral aspects related to the COVID-19 epidemic in France. The survey received approval from the CNIL (French Data Protection Authority) on April 25, 2020 (ref: MLD/MFI/AR205138) and the “Comité de protection des personnes” (the French equivalent of the Research Ethics Committee) on April 24, 2020. It also obtained certification from the “Comité du Label de la statistique publique,” ensuring its adherence to statistical quality standards.

The cohort protocol, detailed in another publication (11), involved a random sample of 135,000 individuals aged 15 and over, drawn from the tax database of the National Institute of Statistics and Economic Studies (INSEE). This database covers 96% of the population living in France, excluding those in institutional settings. The first wave of the study occurred in May 2020 when COVID-19 tests were not widely available. A second wave was conducted in November and December 2020, which included questions on access to testing. In total, 107,808 respondents participated in this second wave, representing 81.7% of the first wave participants. Respondents completed the questionnaire online, with phone interviews available for those without internet access. Additionally, 10% of internet-accessible participants were randomly selected for phone interviews to account for potential method collection effects.

**Figure 1:**
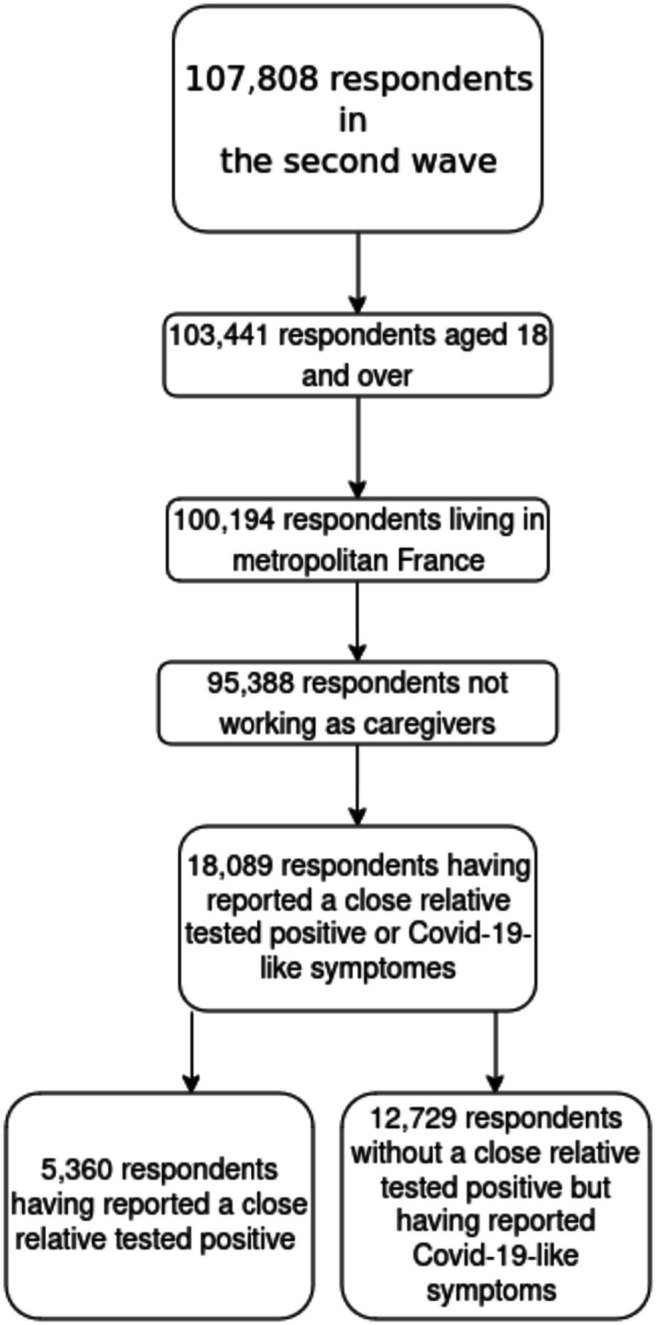
Sample selection.

This study is based on the second wave of EpiCoV, including 107,808 respondents. We excluded individuals aged 17 or younger (4,544 observations). We also excluded individuals living in overseas territories (3,247 observations) due to significant differences in healthcare systems with metropolitan France (continental France and Corsica). Caregivers were also excluded (4,806 observations) as they could be tested at their workplace. The final sample comprised 95,388 individuals (see Figure 1).

### Outcome

Participants were asked the following question: “Have you been tested for the coronavirus with a nasal swab or a saliva test?” with response options: “Yes, once/Yes, more than once/Never.” Therefore, the outcome variable should be interpreted as “the participant has already been tested at least once since the beginning of the pandemic.”

### Socio-economic variables

We considered six socio-economic variables: sex, age, education, income, and ethno-racial status. Sex was a binary variable (male/female). Age was categorized into seven brackets: 18–24, 25–34, 35–44, 45–54, 55–64, 65–74, and 75 or older. Education levels were measured according to the hierarchical grid of diplomas in France: no degree, professional degree, completed high school, college degree, and higher degree. Income deciles were obtained from fiscal data matched with the sample. Ethno-racial status distinguished the mainstream population (individuals residing in metropolitan France who are neither immigrants nor natives of French Overseas Departments, nor descendants of immigrants or DOM natives) from minority populations, with distinctions between first-generation (immigrants) and second-generation (descendants of immigrants) based on country of origin. The term “racialized” refers to individuals from the Maghreb, Turkey, Asia, and Sub-Saharan Africa.

### Exposure risk factors

We considered five main risk factors for COVID-19 exposure:

1 Household size: single, 2, 3, or 4 or more persons.
2 Population density: defined by Eurostat methodology with urban centers (areas with more than 50,000 inhabitants and a population density of at least 1,500 inhabitants per km^2^) and urban clusters (areas with more than 5,000 inhabitants and a population density of at least 350 inhabitants per km^2^). The survey contains information about the city of residence of each participant, and this information was matched with outside information from INSEE about the density of each city (more precisely, each “commune”) in France.
3 Workplace exposure: respondents reported their work status during the 7 days before the interview (not worked, worked only from home, worked both from home and outside, or worked exclusively outside). Since this question was asked during both the first and second waves of the survey, we use the responses collected from both waves. We therefore categorized this variable in four categories:

1. those who had not worked during any wave;
2. those who worked exclusively from home during at least one of the two waves;
3. those who worked part time outside home during at least one of the two waves;
4. those who have worked full time outside home during both waves.

4 Geographical variations in prevalence: regions were categorized as Grand Est, Hauts-de-France, Ile-de-France, Auvergne-Rhône-Alpes, Bourgogne-Franche-Comté, Provence-Alpes-Côte d’Azur, and less exposed regions.
5 Chronic disease: respondents indicated whether they had at least one chronic disease.

### Statistical methods

Statistical analysis was adjusted using weights established by INSEE to produce representative estimators of the population. We focused on two high-risk situations and stratified the sample accordingly. The first stratum included individuals who responded affirmatively to the question: “Since the beginning of the epidemic (February 2020), has another person living with you tested positive for the coronavirus through a nasal swab or a blood test (antibodies)?” This group comprised 5,360 individuals. The second stratum included 12,729 individuals who reported experiencing COVID-19-like symptoms, defined by the European Centre for Disease Prevention as at least one of the following: cough, fever, dyspnea, or sudden onset of ageusia, dysgeusia, or anosmia, between July 1 and August 21, 2022, or after September 1 (Table 1).

**Table 1:** Descriptive statistics of the stratified samples.

	Close positive (*N* = 5,360)	Symptoms (*N* = 12,729)
Sex		
Male	50% (2,635)	44% (5,262)
Female	50% (2,725)	56% (7,467)
Missing	0% (0)	0% (0)
Age		
18–24	16% (780)	16% (1,912)
25–34	14% (612)	22% (2,385)
35–44	16% (916)	20% (2,823)
45–54	23% (1,432)	16% (2,402)
55–64	16% (972)	12% (1,786)
65–74	9% (497)	8% (1,046)
75+	5% (151)	5% (375)
Missing	0% (0)	0% (0)
Education		
No degree	20% (610)	15% (1,164)
Professional degree	19% (982)	16% (1,970)
Completed High School	23% (1,212)	24% (2,996)
College	24% (1,576)	28% (4,048)
Master or more	14% (980)	17% (2,551)
Missing	0% (0)	0% (0)
Income decile		
D1	8% (369)	9% (1,083)
D2–D3	18% (685)	17% (1,733)
D4–D5	18% (794)	19% (1,969)
D6–D7	18% (999)	20% (2,564)
D8–D9	22% (1,441)	21% (3,292)
D10	12% (942)	10% (1,709)
Missing	4% (130)	5% (379)
Ethno-racial status		
Born in metropolitan France from French born parents	70% (4,072)	75% (9,973)
Born in French overseas territories from French born parents	2% (91)	2% (216)
Racialised first generation immigrants	8% (270)	6% (490)
Non-racialised first generation immigrants	4% (142)	3% (313)
Racialised second generation immigrants	6% (273)	6% (625)
Non-racialised second generation immigrants	5% (299)	5% (648)
Missing	5% (213)	4% (464)
Nb of persons in household		
Single	0% (0)	23% (2,499)
2 person	36% (1,846)	30% (3,822)
3 persons	23% (1,285)	19% (2,484)
4 persons or more	41% (2,229)	29% (3,924)
Missing	0% (0)	0% (0)
Population density		
Urban center	45% (2,231)	41% (4,870)
Urban cluster	26% (1,479)	28% (3,652)
Less dense area	28% (1,650)	31% (4,207)
Missing	0% (0)	0% (0)
Region		
Least exposed regions	27% (1,543)	36% (4,620)
Grand Est	9% (559)	8% (1,178)
Hauts-de-France	11% (599)	10% (1,260)
Ile-de-France	27% (1,296)	21% (2,511)
Auvergne-Rhone-Alpes	15% (769)	13% (1,748)
Bourgogne-Franche-Comte	4% (238)	4% (536)
Provence-Alpes-Cotes d’Azur	7% (356)	8% (876)
Missing	0% (0)	0% (0)
Workplace		
Has not worked	41% (1,877)	38% (4,377)
Only remote working	9% (532)	8% (1,187)
Part time work outside home	42% (2,432)	45% (6,005)
Full time work outside home	9% (502)	9% (1,120)
Missing	<0.5% (17)	<0.5% (40)
Symptoms		
No symptom	57% (2,971)	0% (0)
Symptoms before July 1	14% (775)	0% (0)
Symptoms July 1–August 31	2% (110)	12% (1,493)
Symptoms after September 1	27% (1,499)	88% (11,236)
Missing	<0.5% (5)	0% (0)
Chronic disease		
No	67% (3,691)	63% (8,240)
Yes	33% (1,668)	37% (4,485)
Missing	<0.5% (1)	<0.5% (4)

The socio-economic and risk exposure variables selected were those who showed some association with risk of infection or protective behavior in previous studies (12, 13).

We first describe the socio-economic and health characteristics of those tested, for each stratified subsample. The share of individuals who go tested was estimated for each subsample, with the associated 95% confidence interval. Logistic regressions were then performed to identify characteristics associated with testing, independent of other variables. We first estimated crude odds-ratios for each variable, and then performed a multinomial regression to estimate adjusted odds-ratios. Tables 2, 3 below report these estimates, with their 95% confidence interval and the associated *p*-value.

**Table 2:** COVID-19 testing for individuals who have experienced COVID-19-like symptoms but did not report having a close relative test positive (*N* = 12,729).

Variable	Testing Freq (95% CI)	Crude OR	(95% CI)	*p*-value	Adjusted OR	(95% CI)	*p*-value
Sex							
Male	44% (42–46%)	ref			ref		
Female	47% (46–49%)	1.03	[1.02–1.05]	<0.01	1.04	[1.01–1.06]	<0.01
Age							
18–24	53% (50–56%)	1.12	[1.08–1.15]	<0.01	1.14	[1.09–1.18]	<0.01
25–34	49% (46–51%)	1.06	[1.03–1.09]	<0.01	1.04	[1.00–1.07]	0.05
35–44	46% (43–48%)	1.02	[1.00–1.05]	0.09	1.03	[0.99–1.06]	0.14
45–54	43% (40–45%)	ref			ref		
55–64	43% (40–46%)	1.00	[0.97–1.03]	0.89	1.00	[0.96–1.05]	0.85
65–74	38% (34–41%)	0.95	[0.91–0.98]	<0.01	0.97	[0.92–1.02]	0.24
75+	41% (34–47%)	0.98	[0.93–1.04]	0.53	0.99	[0.92–1.07]	0.84
Education							
No degree	42% (38–45%)	0.97	[0.93–1.00]	0.05	1.02	[0.98–1.07]	0.30
Professional degree	40% (37–42%)	0.96	[0.93–0.99]	<0.01	1.00	[0.97–1.04]	0.87
Completed High School	44% (42–46%)	ref			ref		
College	48% (46–50%)	1.02	[0.99–1.04]	0.21	1.04	[1.01–1.07]	<0.01
Master or more	54% (51–56%)	1.08	[1.05–1.11]	<0.01	1.08	[1.04–1.12]	<0.01
Income decile							
D1	42% (38–46%)	ref			ref		
D2–D3	45% (42–48%)	1.03	[0.99–1.07]	0.11	1.03	[0.98–1.08]	0.20
D4–D5	43% (41–46%)	1.02	[0.98–1.06]	0.27	1.02	[0.98–1.07]	0.36
D6–D7	47% (44–49%)	1.04	[1.00–1.08]	0.03	1.05	[1.00–1.10]	0.03
D8–D9	47% (45–49%)	1.04	[1.01–1.08]	0.02	1.05	[1.00–1.09]	0.05
D10	51% (48–53%)	1.08	[1.04–1.12]	<0.01	1.07	[1.02–1.13]	0.01
Ethno-racial status							
Born in metropolitan France from French born parents	45% (44–46%)	ref			ref		
Born in French overseas territories from French born parents	52% (44–60%)	1.07	[1.00–1.15]	0.04	1.06	[0.97–1.15]	0.19
Racialised first generation immigrants	44% (38–49%)	1.00	[0.95–1.04]	0.89	0.99	[0.93–1.05]	0.69
Non-racialised first generation immigrants	47% (40–54%)	1.03	[0.97–1.09]	0.28	1.01	[0.95–1.09]	0.71
Racialised second generation immigrants	52% (47–57%)	1.07	[1.03–1.12]	<0.01	1.04	[0.99–1.10]	0.13
Non-racialised second generation immigrants	45% (40–49%)	1.00	[0.97–1.05]	0.82	1.00	[0.95–1.05]	0.92
Nb of persons in household							
Single	47% (45–50%)	1.04	[1.01–1.07]	<0.01	1.02	[0.99–1.05]	0.28
2 persons	45% (43–47%)	ref			ref		
3 persons	47% (45–50%)	1.03	[1.00–1.06]	0.03	1.00	[0.97–1.04]	0.81
4 persons or more	44% (43–46%)	0.99	[0.97–1.02]	0.62	0.97	[0.94–1.00]	0.08
Population density							
Urban center	50% (48–51%)	1.10	[1.07–1.12]	<0.01	1.05	[1.02–1.08]	<0.01
Urban cluster	45% (43–47%)	1.04	[1.02–1.07]	<0.01	1.02	[0.99–1.05]	0.15
Less dense area	41% (40–43%)	ref			ref		
Workplace							
Has not worked	42% (40–44%)	ref			ref		
Only remote working	54% (50–57%)	1.09	[1.06–1.13]	<0.01	1.07	[1.03–1.12]	<0.01
Part time work outside home	48% (47–50%)	1.06	[1.04–1.08]	<0.01	1.06	[1.03–1.09]	<0.01
Work outside home	42% (39–46%)	1.01	[0.98–1.04]	0.55	1.03	[0.99–1.08]	0.19
Date of symptoms							
Between July 1 and August 31	52% (49–56%)	1.10	[1.07–1.13]	<0.01	1.08	[1.05–1.12]	<0.01
After September 1	45% (44–46%)	ref			ref		
Chronic disease							
No	46% (45–48%)	ref			ref		
Yes	45% (43–47%)	0.98	[0.97–1.00]	0.08	1.03	[1.00–1.05]	0.03

**Table 3:** COVID-19 testing for individuals who reported having a close relative test positive (*N* = 5,360).

Variable	Testing Freq (95% CI)	Crude OR	(95% CI)	*p*-value	Adjusted OR	(95% CI)	*p*-value
Sex							
Male	60% (57–62%)	ref			ref		
Female	61% (59–63%)	1.03	[1.01–1.06]	0.02	1.00	[0.97–1.03]	0.88
Age							
18–24	64% (60–68%)	1.05	[1.01–1.10]	0.02	1.05	[0.99–1.10]	0.11
25–34	65% (61–70%)	1.05	[1.00–1.10]	0.06	1.03	[0.97–1.09]	0.29
35–44	64% (61–68%)	1.06	[1.01–1.10]	0.01	1.04	[0.99–1.09]	0.16
45–54	61% (58–64%)	ref			ref		
55–64	58% (54–62%)	0.97	[0.93–1.01]	0.15	1.00	[0.95–1.06]	0.95
65–74	55% (49–61%)	0.99	[0.94–1.04]	0.65	1.01	[0.94–1.09]	0.78
75+	42% (32–52%)	0.86	[0.79–0.93]	<0.01	0.93	[0.84–1.03]	0.16
Education							
No degree	55% (50–60%)	0.96	[0.91–1.01]	0.09	0.99	[0.93–1.05]	0.63
Professional degree	57% (53–61%)	0.97	[0.93–1.01]	0.12	1.00	[0.95–1.05]	0.98
Completed High School	61% (58–65%)	ref			Ref		
College	63% (61–66%)	1.01	[0.97–1.05]	0.70	1.02	[0.98–1.06]	0.39
Master or more	65% (62–69%)	1.03	[0.99–1.07]	0.17	1.02	[0.97–1.08]	0.37
Income decile							
D1	61% (54–67%)	ref			Ref		
D2–D3	59% (55–64%)	1.01	[0.95–1.08]	0.74	0.98	[0.91–1.06]	0.70
D4–D5	60% (55–64%)	1.00	[0.94–1.06]	0.94	1.01	[0.94–1.09]	0.81
D6–D7	61% (57–65%)	1.03	[0.97–1.10]	0.27	1.02	[0.95–1.10]	0.52
D8–D9	60% (57–63%)	1.00	[0.95–1.06]	0.89	1.02	[0.95–1.09]	0.68
D10	62% (59–65%)	1.02	[0.96–1.08]	0.52	1.04	[0.96–1.12]	0.33
Ethno-racial status							
Born in metropolitan France from French born parents	60% (58–61%)	ref			ref		
Born in French overseas territories from French born parents	52% (40–64%)	0.97	[0.87–1.07]	0.53	0.92	[0.82–1.04]	0.18
Racialised first generation immigrants	69% (62–75%)	1.07	[1.01–1.14]	0.02	1.10	[1.02–1.19]	0.01
Non-racialised first generation immigrants	54% (43–64%)	0.98	[0.90–1.07]	0.71	0.98	[0.88–1.08]	0.66
Racialised second generation immigrants	67% (60–74%)	1.08	[1.02–1.15]	0.01	1.05	[0.97–1.12]	0.22
Non-racialised second generation immigrants	57% (50–64%)	0.97	[0.91–1.02]	0.26	0.98	[0.92–1.05]	0.56
Nb of persons in household							
2 persons	56% (53–59%)	ref			ref		
3 persons	63% (60–66%)	1.07	[1.03–1.11]	<0.01	1.04	[1.00–1.09]	0.06
4 persons or more	63% (60–65%)	1.06	[1.03–1.10]	<0.01	1.03	[0.98–1.07]	0.21
Population density							
Urban center	63% (60–66%)	1.08	[1.04–1.11]	<0.01	1.05	[1.01–1.10]	0.02
Urban cluster	61% (57–64%)	1.05	[1.01–1.09]	0.01	1.05	[1.01–1.09]	0.02
Less dense area	56% (53–59%)	ref			ref		
Workplace							
Has not worked	56% (53–59%)	ref			ref		
Only remote working	66% (61–71%)	1.08	[1.03–1.14]	<0.01	1.02	[0.96–1.09]	0.50
Part time work outside home	63% (61–65%)	1.04	[1.01–1.07]	0.02	1.04	[0.99–1.08]	0.09
Work outside home	60% (54–65%)	1.01	[0.97–1.07]	0.57	1.03	[0.97–1.10]	0.36
Symptoms							
No symptom	53% (50–55%)	ref			ref		
Symptoms before July 1	46% (41–50%)	0.90	[0.86–0.93]	<0.01	0.94	[0.89–0.99]	0.01
Symptoms July 1–August 31	73% (63–84%)	1.24	[1.14–1.35]	<0.01	1.21	[1.08–1.35]	<0.01
Symptoms after September 1	83% (80–85%)	1.32	[1.29–1.36]	<0.01	1.34	[1.30–1.39]	<0.01
Chronic disease							
No	62% (60–63%)	ref			ref		
Yes	58% (55–61%)	0.98	[0.95–1.00]	0.10	0.99	[0.95–1.02]	0.45

Observations with missing values on key socio-economic and exposure variables were excluded from the multivariable analysis (in all 15%). All analyses were conducted using R software (version 4.2.3). Data wrangling was performed with the help of the tidyverse (14), weighted analyses with the survey package (15) and some summaries were produced using the gtsummary package (16). Percentages presented are weighted to account for the sample design. Multivariable analyses are also weighted. Given the sample size, observed differences were consistently statistically significant, so no tests are presented for univariate analyses (Figure 1).

## Results

The following table provides descriptive statistics for the two stratified samples. For each variable category, we present the share in the weighted sample, the unweighted number of observations, and the 95% confidence interval (CI) of this share. Additionally, for each variable, we include the (unweighted) number of missing values and their (weighted) share with the associated confidence interval.

Tables 2, 3 below present the results of the regression analysis. The outcome variable is coded as 1 if the individual has undergone testing. For each variable, the tables display the weighted proportion of individuals who have been tested along with the 95% confidence interval (CI), the associated odds ratio with its CI, and the *p*-value. This information is provided for both the univariate and multivariable regression analyses. The multivariable analysis also includes controls for the region of residence, which are detailed in the appendix.

Among the 12,729 individuals who had no close relative test positive but reported COVID-19-like symptoms after July 1, 2020, 45.8% underwent testing. Women were more likely than men to get tested, with an odds ratio (OR) of 1.04 (95% CI [1.01–1.06], *p* < 0.01) in the multivariable analysis. Testing likelihood also decreased with age up to 35 years: compared to those aged 45–55 years, individuals aged 18–24 were 1.14 (95% CI [1.09–1.18]) times more likely to test, and those aged 25–34 were 1.04 (95% CI [1.00–1.07]) times more likely to test. Education level was positively associated with testing: compared to those who only completed high school, individuals with a college degree were 1.04 (95% CI [1.01–1.07]) times more likely to test, and those with a master’s degree were 1.08 (95% CI [1.04–1.12]) times more likely to test. Testing was also positively associated with income: compared to individuals in the lowest income decile, those in the sixth decile or above were 1.05 times more likely to test (95% CI [1.00–1.09]). No significant difference in testing was observed based on the country of origin once income and education were controlled for. Living in a household with four or more persons was negatively associated with testing (OR = 0.97, 95% CI [0.94–1.00] compared to households with two persons). Living in an urban center was associated with a higher likelihood of testing compared to living in a low-density area (OR = 1.05, 95% CI [1.02–1.08]). Individuals who worked exclusively from home during the first and second waves of the survey were more likely to test (OR = 1.07, 95% CI [1.03–1.12]), as were those who worked partly from home (OR = 1.06, 95% CI [1.03–1.09]). The timing of symptoms also played a role, with those experiencing symptoms before August 31 being 1.08 times more likely to test (95% CI [1.05–1.12]). Finally, suffering from a chronic disease slightly increased the likelihood of testing (OR = 1.03, 95% CI [1.00–1.05]).

Overall among the 5.360 individuals who had a close relative test positive, approximately 60% had been tested. There was no significant difference in testing likelihood between women and men. When controlling for all variables, the multivariate regression did not reveal any significant effects of age, income, or education on the likelihood of testing (at the 5% significance level).

However, compared to individuals born in metropolitan France to French parents, racialized first-generation immigrants were 1.10 times more likely to be tested (95% CI [1.02–1.19]). Individuals living in urban areas (either centers or clusters) were also more likely to be tested, with odds ratios of 1.05 (95% CI [1.00–1.10] for centers) and 1.05 (95% CI [1.00–1.09] for clusters). Living in a three-person household increased the likelihood of testing by 1.04 times (95% CI [1.00–1.09]) compared to living in a two-person household.

Among those who had a close relative test positive and also experienced symptoms, testing likelihood varied with the timing of symptoms. Individuals who experienced symptoms before July 1 were less likely to test compared to those with no symptoms (OR = 0.94, 95% CI [0.89–0.99]). Conversely, those who experienced symptoms between July 1 and August 31 were 1.21 times more likely to test (95% CI [1.08–1.35]), and those with symptoms after September 1 were 1.34 times more likely to test (95% CI [1.30–1.39]). Notably, among individuals who had a close relative test positive and experienced symptoms after September 1, 83% underwent testing. This sharp increase in testing rates during the summer of 2020 reflects the transition from a period of limited testing availability to widespread access.

## Discussion

Our analysis of testing behavior, based on a representative random survey with detailed data on socio-economic variables and exposure to COVID-19 risk, reveals that the association between socio-economic conditions and testing behavior varies across different risk situations.

In the first scenario, where individuals have experienced symptoms but no household member has tested positive, we found that women were more likely than men to get tested. Testing was positively associated with being younger, wealthier, more educated, and living in an urban area. This aligns with previous studies (5, 7–9) which utilized administrative data and ecological variables to find similar trends.

Conversely, in the second scenario, where a household member had tested positive, the likelihood of testing was much higher overall, but in this case, the only socio-economic variable positively associated with testing was population density. Testing was strongly correlated with having experienced symptoms, particularly when symptoms occurred during periods of increased testing availability. These findings underscore the value of survey data in complementing administrative sources and highlight the importance of considering exposure risk when analyzing socio-economic inequalities in testing.

Nevertheless, this study has some limitations. First, while we observe socio-economic conditions, exposure to risk, and testing behavior at the individual level, we lack precise data on the timing of exposure and testing. We cannot determine whether a test was taken before or after a higher risk situation. Second, the survey collected only proxy information on ethno-racial status. Given that individuals from ethno-racial minorities are more likely to work in high-risk jobs (13), exploring this dimension could provide additional insights. Third, like many national population-based surveys, our study does not capture highly vulnerable groups such as undocumented migrants and homeless individuals, who are particularly affected by the pandemic (13). Additionally, people living in institutions like nursing homes were excluded from the sampling design. Finally, since the outcome under consideration is quite frequent in each subsample, adjusted odds-ratios may overestimate the association (17, 19).

Given these limitations, our findings should be interpreted with caution. Although we refrain from causal interpretations, some mechanisms can be suggested. As Bevan et al. (18) highlighted, a key motivation for testing is a sense of solidarity—testing to protect others by isolating oneself if the test result is positive. This motivation is stronger when the individuals needing protection are household members. When no close relative has tested positive, an individual testing positive risks introducing the virus to their household, which may increase their motivation to test. In contrast, if a close relative has already tested positive, isolation may be less effective as other family members might have already been exposed. Therefore, the motivation to test is likely stronger in the symptom-only scenario compared to the close-contact scenario.

Isolation also requires resources. Wealthier individuals are more likely to have the means to isolate themselves, such as living in larger homes or owning secondary properties. Lower-income individuals, who often live in more crowded conditions, may find it more challenging to isolate themselves, reducing the incentive to test. Additionally, Bajos et al. (12) found that social discrimination can weaken solidarity motives in vaccination, which could also apply to testing. Since poorer and less educated individuals experience more discrimination, this might further explain their lower testing rates. In summary, socio-economic status influences not only the ability to act on a positive test result, but also the solidarity motive for testing.

Other findings are more straightforward. Population density is positively associated with testing, possibly due to closer proximity to testing facilities in urban areas compared to rural ones. The positive association between education and testing in the “symptoms” group may reflect greater awareness and adherence to health recommendations. Additionally, having a close relative test positive may increase one’s awareness of infection risk.

Overall, our study provides moderate evidence that testing is associated with socio-economic variables. While the multivariable analysis showed no significant links between testing and income, education, socio-professional group, or country of origin in the “close contact” group, socio-economic status still influenced testing behavior in the “symptoms” group. This suggests that even if tests were available at no financial cost, socio-economic inequalities in access to testing were not entirely eliminated.

## Generalizability

France’s approach to free COVID-19 testing differentiates it from many other countries where individuals incur out-of-pocket costs. This unique aspect of the French context may limit the generalizability of our findings to other countries. While testing for other infections, such as HIV or hepatitis, is also free in France in some cases without a medical prescription, the specific visibility, awareness, and susceptibility associated with COVID-19 might lead to different patterns for other infectious diseases.

## Data Availability

The data analyzed in this study is subject to the following licenses/restrictions: available on request to the Epicov team. Requests to access these datasets should be directed to frederic.robergeau@inserm.fr.

## Footnotes

^1^ https://ourworldindata.org/coronavirus-testing#how-many-tests-are-performed-each-day

## The EPICOV study group

The members of EPICOV study group: Nathalie Bajos (co-principal investigator), Josiane Warszawski (co-principal investigator), Guillaume Bagein, Emilie Counil, Florence Jusot, Claude Martin, Laurence Meyer, Philippe Raynaud, Alexandra Rouquette, Ariane Pailhé, Delphine Rahib, Patrick Sicard, Alexis Spire.

## References

[1] World Health Organization. Laboratory testing for coronavirus disease 2019 (COVID-19) in suspected human cases. Interim guidance. (2020). Available at: https://iris.who.int/bitstream/handle/10665/331329/WHO-COVID-19-laboratory-2020.4-eng.pdf?sequence=1&isAllowed=y (Accessed December 27, 2024).

[2] Richard Z Chabrol F Gautier L Zinszer K Ridde V. Considering social inequalities in health in COVID-19 response: insights from a French case study. Health Promot Int. (2023) 38:1–11. doi: 10.1093/heapro/daac173, PMID: 36617297

[3] Savard A Gabet M Richard Z Oliveira SRDA Coulibaly A Cazarin G. A descriptive comparison of mass testing during the COVID-19 pandemic in Montreal, Paris, Bamako, and Recife. Int J Public Health. (2022) 67:1604992. doi: 10.3389/ijph.2022.1604992, PMID: 36213140 PMC9537363

[4] Raeesi A Kiani B Hesami A Goshayeshi L Firouraghi N Ebrahimi SM. Access to the COVID-19 services during the pandemic - a scoping review. Geospat Healt. (2022) 17. doi: 10.4081/gh.2022.1079, PMID: 35352541

[5] Green MA García-Fiñana M Barr B Burnside G Cheyne CP Hughes D. Evaluating social and spatial inequalities of large scale rapid lateral flow SARS-CoV2 antigen testing in COVID-19 management: an observational study of Liverpool, UK (November 2020 to January 2021). Lancet Reg Health Europe. (2021) 6:100107. doi: 10.1016/j.lanepe.2021.100107, PMID: 34002172 PMC8114854

[6] Baggio S Jacquerioz F Salamun J Spechbach H Jackson Y. Equity in access to COVID-19 testing for undocumented migrants and homeless persons during the initial phase of the pandemic. J Migrat Health. (2021) 4:100051. doi: 10.1016/j.jmh.2021.100051, PMID: 34184000 PMC8214821

[7] Dryden-Peterson S Velásquez GE Stopka TJ Davey S Lockman S Ojikutu BO. Disparities in SARS-CoV2 testing in Massachusetts during the COVID-19 pandemic. JAMA Netw Open. (2021) 4:e2037067. doi: 10.1001/jamanetworkopen.2020.37067, PMID: 33560423 PMC7873783

[8] Riou J Panczak R Althaus CL Junker C Perisa D Schneider K. Socioeconomic position and the COVID-19 care cascade from testing to mortality in Switzerland: a population-based analysis. Lancet Public Health. (2021) 6:e683–91. doi: 10.1016/S2468-2667(21)00160-2, PMID: 34252364 PMC8270761

[9] Vandentorren S Smaïli S Chatignoux E Maurel M Alleaume C Neufcourt L. The effect of social deprivation on the dynamic of SARS-CoV2 infection in France: a population-based analysis. Lancet Public Health. (2022) 7:e240–9. doi: 10.1016/S2468-2667(22)00007-X, PMID: 35176246 PMC8843336

[10] Trevisan C Pedone C Maggi S Noale M di M Sojic A. Accessibility to SARS-CoV-2 swab test during the COVID-19 pandemic: did age make the difference? Health Policy. (2021) 125:1580–6. doi: 10.1016/j.healthpol.2021.10.002, PMID: 34649753 PMC8492891

[11] Warsawski J Bagein G Derohyon T Bajos N EpiCoV study group. EpiCoV: a large population-based cohort in COVID-19 times: methodological issues. Journées de méthodologie statistique de l’INSEE. (2022) 14, 1–28.

[12] Bajos N Spire A Silberzan L Sireyjol A Jusot F Meyer L. When lack of trust in the government and in scientists reinforces social inequalities in vaccination against COVID-19. Front Public Health. (2022) 10:908152. doi: 10.3389/fpubh.2022.908152, PMID: 35937246 PMC9346080

[13] Gosselin A Warszawski J Bajos N EpiCoV Study Group. Higher risk, higher protection: COVID-19 risk among immigrants in France-results from the population-based EpiCoV survey. Eur J Pub Health. (2022) 32:655–63. doi: 10.1093/eurpub/ckac046, PMID: 35478253 PMC9341671

[14] Wickham H Averick M Bryan J Chang W McGowan L François R. Welcome to the tidyverse. J Open Source Softw. (2019) 4:1686. doi: 10.21105/joss.01686

[15] Lumley T. Complex surveys: a guide to analysis using R. (2010). Wiley, Hoboken, New Jersey, USA.

[16] G/Daniel D Sjoberg Whiting K Curry M G/Jessica J Lavery. Reproducible summary tables with the gtsummary package. R J. (2021) 13:570–80. doi: 10.32614/RJ-2021-053

[17] Barros AJ Hirakata VN. Alternatives for logistic regression in cross-sectional studies: an empirical comparison of models that directly estimate the prevalence ratio. BMC Med Res Methodol. (2003) 3:21. doi: 10.1186/1471-2288-3-21, PMID: 14567763 PMC521200

[18] Bevann I Ba14xter MS Stagg HR Street A. Knowledge, attitudes, and behavior related to COVID-19 testing: a rapid scoping review. Diagnostics (Basel). (2021) 11:1685. doi: 10.3390/diagnostics11091685, PMID: 34574026 PMC8472251

[19] Yorlets RR Lee Y Gantenberg JR. Calculating risk and prevalence ratios and differences in R: developing intuition with a hands-on tutorial and code. Ann Epidemiol. (2023) 86:104–9. doi: 10.1016/j.annepidem.2023.08.001. Erratum in: Ann Epidemiol. 2024;99:47. doi: 10.1016/j.annepidem.2024.09.003, PMID: 37572803

